# 

**Published:** 1879-07

**Authors:** 


					﻿JULY, 1879.
TWENTY-SIXTH YEAR—No 307.
HALL’S
Journal of Health.
PUBLISHED AT 141 EIGHTH STREET,
(Near Broadway),	INTJETW" YORK.
E. H. GIBBS, A.M., M.D., Editor.
AGENTS EOK THE TRADE:
AMERICAN NEWS COMPANY AND NEW YORK NEWS COMPANY
Terms, $1.50 a Year, or $1.00 for Eight Months. Postage paid.
SINGLE NUMBER, 15 CENTS.
Couch & Johmbtox, Printers, 11 Frank! ort Street, New York.
				

